# The epidemiology and mutation types of Leber’s hereditary optic neuropathy in Thailand

**DOI:** 10.1080/07853890.2022.2082517

**Published:** 2022-06-06

**Authors:** Kanchalika Sathianvichitr, Benjaporn Sigkaman, Niphon Chirapapaisan, Poramaet Laowanapiban, Tanyatuth Padungkiatsagul, Supanut Apinyawasisuk, Juthamat Witthayaweerasak, Wanicha Chuenkongkaew

**Affiliations:** a Department of Ophthalmology, Faculty of Medicine Siriraj Hospital, Mahidol University, Bangkok, Thailand; b Department of Ophthalmology, Bhumibol Adulyadej Hospital, Bangkok, Thailand; c Ophthalmology Service, Mettapracharak (Wat Rai Khing) Hospital, Nakhon Pathom, Thailand; d Department of Ophthalmology, Faculty of Medicine Ramathibodi Hospital, Mahidol University, Bangkok, Thailand; e Department of Ophthalmology, Faculty of Medicine, Chulalongkorn University, Bangkok, Thailand; f Ophthalmology Department, King Chulalongkorn Memorial Hospital, Bangkok, Thailand; g Department of Ophthalmology, Faculty of Medicine, Prince of Songkla University, Songkhla, Thailand

**Keywords:** G11778A, T14484C, Leber hereditary optic neuropathy, mitochondrial disease, visual loss

## Abstract

**Purpose:**

Leber’s hereditary optic neuropathy (LHON), the most common mitochondrial optic neuropathy, causes visual loss, especially in young adults. Due to the absence of epidemiological data in Southeast Asia, we aimed to determine Thai LHON patients’ characteristics (demographic data, mutation types, and prognoses) as the first study in this region.

**Methods:**

This retrospective chart review enrolled all Thai LHON patients confirmed by three mitochondrial DNA mutations (G11778A, T14484C, and G3460A) between January 1997 and December 2016. Patients with more than one year of follow-up were included in a visual progression analysis. The Mann–Whitney U-test was applied to compare groups, and prognosis-associated factors were analysed with the generalized estimating equation.

**Results:**

In all, 229 patients were enrolled, with only nineteen females. Most mutations were of the G11778A type (91%), with T14484C accounting for the remainder. The age at onset of G11778A (21.9 years; interquartile range [IQR] 14.9, 33.5) was younger than that of T14484C (33.0 years; IQR 19.4, 37.5). Of 45 patients, the T14484C group demonstrated good vision recovery, whereas the G11778A group did not improve (difference in logMAR −0.7 and IQR −1.5, −0.2 versus logMAR 0.0 and IQR −0.3, 0.2, respectively; *P* value .001). The G11778A mutation, male, and older age were related to poor prognoses.

**Conclusions:**

The leading mutation in Thai LHON patients is the G11778A missense, followed by T14484C, while G3460A was not detected. The vast majority of patients were young adult males. The G11778A mutation, older age, and male gender are associated with poor vision outcomes.

**Key message**
- The G11778A missense mutation is the most common among Thai LHON patients, followed by T14484C, while G3460A was not found. The G11778A mutation, older age, and male gender are associated with poor vision outcomes.

## Introduction

Leber’s hereditary optic neuropathy (LHON) is the most common mitochondrial optic neuropathy. Its prevalence ranges across regions, with 1:30,000 to 1:54,000 in Europe [1–4] and a much lower proportion of 1:68,403 in Australia [5]. The classic manifestation is the subacute painless central visual loss of the of both eyes of young adult males. The visual loss may occur sequentially or, less frequently, simultaneously. Although its pathognomonic feature is a hyperaemic optic disc with surrounding telangiectasia, this sign is unlikely to show in an eye examination because it appears only briefly during the acute phase. More frequently, a temporally pale disc is the only sign that remains. This non-specific finding makes diagnosis challenging.

In recent years, genetic testing has played a crucial role in LHON diagnosis. The testing focuses on three common mutations, G11778A, T14484C, and G3460A, which account for more than 95% of cases [6]. These missense mutations occur in mitochondrial DNA (mtDNA). They code a subunit of the respiratory chain complex I, leading to free radical accumulation and cell apoptosis [6]. Another positive attribute of genetic testing is its prognostic capability. While the G11778A mutation produces the most severe visual loss with the poorest recovery, the visual loss caused by T14484C has the best chance of recovery. In contrast to other mitochondrial diseases, maternal inheritance in LHON patients manifest incomplete penetration and a male predominance. The reasons for this are not well established. Several studies [7–9] proposed that environmental factors such as smoking and alcohol consumption, hormonal factors and genetic modifiers including secondary mutations and haplogroup status are involved.

Though the prevalence, genetic mutations, and characteristics of LHON patients vary among ethnicities, one similarity is shared: the G11778A mutation accounts for the largest proportion of LHON cases, followed by the T14484C mutation. Two studies on LHON patients from Southeast Asia [10,11] found that the genetic details of the G11778A mutation differed from those in European and Japanese LHON patients. Nevertheless, there is currently no epidemiological data relating to LHON patients in Thailand nor in Southeast Asia more broadly. This study set out to establish epidemiological data for the Thai population. The research was conducted at the Faculty of Medicine, Siriraj Hospital, Mahidol University. This institution is not only the largest tertiary-care referral hospital in Thailand, but it was also the only centre performing LHON-genetic testing in the nation during the 1997–2016 study period. The current investigation was therefore able to enrol all LHON patients confirmed by genetic diagnosis in Thailand during that period.

## Patients and methods

### Patients

This retrospective chart review was conducted in accordance with the Declaration of Helsinki at the Department of Ophthalmology, Faculty of Medicine Siriraj Hospital, Thailand. The study recruited all patients diagnosed with LHON between January 1997—when the first case was confirmed genetically—and December 2016. Before commencement of this research, its protocol was approved by the Siriraj Institutional Review Board, Faculty of Medicine Siriraj Hospital, Mahidol University, Bangkok, Thailand.

The clinical diagnoses were based on a combination of symptoms and signs. All patients presented with subacute, painless, bilateral visual loss in either a simultaneous or a sequential pattern. The optic disc showed hyperaemia with circumpapillary telangiectatic vessels. Central or cecocentral scotoma was detected by automated perimetry. Family histories were based on patient interviews. Highly suspicious patients were still included even if they had a negative family history.

**Figure 1.**
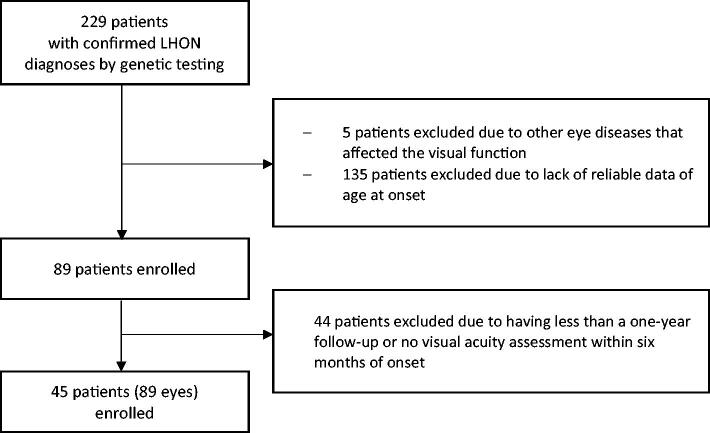
Flow diagram of patient selection process.

Patients with any other eye diseases that could affect vision were excluded. To conduct visual progression analyses, we selected patients whose (1) best-corrected visual acuity (BCVA) was measured within 6 months of LHON onset; and (2) follow-up periods exceeded one year (Figure 1).

### Data collection

We reviewed the patients’ medical records to collect their demographic, genetic-testing, and clinical data. The clinical component comprised details of the onset of blurred vision and the BCVA values at the patients’ first and last visits. Each value was converted to an equivalent number of logMAR (logarithm of the minimal angle of resolution) units. Finger counting at 3 feet, 2 feet, 1 foot, hand motion, and no light perception were also converted to 1.8, 2.0, 2.3, 3, and 4 on the logMAR scale, respectively. The duration of follow-up was calculated in years.

### Molecular genetic testing

Mitochondrial DNA (mtDNA) was extracted from EDTA (ethylenediaminetetraacetic acid) venous blood samples. Before 2013, the mutation at position 11778 was detected with the PCR-RFLP (polymerase chain reaction-restriction fragment length polymorphism) technique. The DNA product was subsequently cut by restriction endonuclease BclI, and the DNA fragments were further run on 4% agarose gel electrophoresis. After 2013, however, the 11778 mutation was analysed by bidirectional PCR, followed by Sanger sequencing. The same technique was also used for the mutations at positions 14484 and 3460. The primer pairs and sequencing primers used by our centre are listed in Table 1.

**Table 1.** PCR and sequencing primer of 3 common genetic tests.

Gene	mtDNA mutation	PCR Primers and sequencing primers (5’→3’)	Temperature (^o^C)	Base position	Product size (bp)
PCR-RFLP					
ND4	G11778A (before 2013)	F: CTC ATT ACT ATT CTG CCT AGC AAA CTC AAA CTA CGA ACG CAC TCA TGA TC R: GTA GGA GAG TGA TAT TTG ATC AGG	55	11728–11942	215
Bi-directional PCR, followed by Sanger sequencing technique					
ND4	G11778A (since 2013)	F: CTC TAC CTC TCT ATA CTA ATC TCC CTA CAA ATC TCC TTA ATG CTA R: GTA GGA GAG TGA TAT TTG ATC AGG S: GTA GGA GAG TGA TAT TTG ATC AGG	55	11039–11942	904
ND6	T14484C	F: CAT CAC CTC AAC CCA AAA AGG C R: GGA TCA GGC AGG CGC CAA GGA GTG S: GGA TCA GGC AGG CGC CAA GGA GTG	55	14061–14873	813
ND1	G3460A	F: CCC GAT GGT GCA GCC GC R: GAG ATT GTT TGG GCT ACT GCT CGC S: TCC TCC CTG TAC GAA AGG AC	55	3007–3728	722

F: forward primer; R: reverse primer; S: sequencing primer.

Limitation of the study. Before 2013, PCR-RFLP was primarily done to detect the G11778A mutation, not the other LHON primary mutation types.

If it did not cause confusion, the S sequencing primer base position should be mentioned.

### Statistical analysis

The mutation ratios and genders are presented as percentages. Means and standard deviations (SD) were calculated for normally distributed continuous data. Non-normally distributed continuous data are reported as medians and interquartile ranges (IQRs). Comparisons were made of the means and medians of the groups of patients with different mutations. The non-parametric Mann–Whitney U-test was applied to ordinal and non-normally distributed continuous data. The unpaired t-test was applied to any continuous data that were normally distributed.

The associations between the final BCVA values and the possibly related factors (point of mutation, visual acuity, age of onset, and gender) were examined by fitting a marginal linear regression model. The generalized estimating equation (GEE) method was used, with each subject serving as a cluster.

The data were prepared and analysed using PASW Statistics for Windows (version 18.0; SPSS Inc., Chicago, IL, USA). A *P* value of <.05 was deemed statistically significant.

## Results

Between January 1997 and December 2016, 229 patients received confirmed diagnoses of LHON, based on genetic testing. Of those patients, 91% (209/229) had a mutation at G11778A; the remaining 20 patients had a mutation at T14484C. No G3460A mutation was found. Only 19 patients (8.3%) were female, and 18 of those had the G11778A mutation. Twenty-two of 209 patients (10.5%) with the G11778A mutation were heteroplasmy and 17 of 22 were male, whereas all T14484 patients were homoplasmy.

To evaluate the age at onset, we enrolled 89 patients (176 eyes) whose onset times could be reliably determined from their medical records. There was no significant difference in the median ages at onset for the G11778A patients (21.9 years; interquartile range [IQR] 14.9, 33.5) and the T14484 patients (33.0 years; IQR 19.4, 37.5). The youngest patient was aged 5 years, while the oldest was 66 years. The demographic data are summarised in Table 2. There was no statistical difference in the median visual acuities at first visit of the G11778A patients (1.8 logMAR units) and T14484C patients (1.5 logMAR units). All patients had colour vision defect. Besides, four patients with G11778A had facioscapulohumeral muscular dystrophy [12].

**Table 2.** Demographic data, by mtDNA mutation.

Mutation position in mtDNA	G11778A *N* = 209 patients	T14484C *N* = 20 patients
Gender		
Male	191 (91.4%)	19 (95%)
Female	18 (8.6%)	1 (5%)
Age at onset	*N* = 79 patients	*N* = 10 patients
Median (years)	21.9	33.0
Range (years)	5.10, 59.93	15.80, 65.90

mt DNA: mitochondrial DNA.

To compare the visual progression of the G11778A and T14484C patients, we extracted the BCVA assessment data relating to 89 eyes. These patients had been examined within 6 months of their respective LHON onsets, and they had been followed up for at least a year from the date of their diagnoses. There was no statistical difference in the median visual acuities at first visit of the G11778A patients (1.6 logMAR units; IQR 1.2, 2.3) and T14484C patients (1.7 logMAR units; IQR 1.0, 2.3). At the last visit, the patients with the T14484C mutation showed considerable recovery of their vision. The visual acuity was improved from 1/60 to 6/19 in the T14484C mutation. In marked contrast, no significant change in the vision of the patients with G11778A was observed (Table 3).

**Table 3.** Comparison of visual progression of LHON patients with G11778A and T14484C mutations whose follow-up exceeded one year.

	Mutation		
	11778 *N* = 79 eyes	14484 *N* = 10 eyes	
	Median (IQR)	Median (IQR)	*p* Value^a^
Follow-up period (years)	5.4 (2.8, 8.3)	2.6 (1.6, 4.5)	***0.004***
BCVA (logMAR) at first visit^b^	1.6 (1.2, 2.3)	1.7 (1.0, 2.3)	0.115
BCVA (logMAR) at last visit	1.6 (1.3, 2.3)	0.5 (0.2, 1.8)	**0.004**
Difference of BCVA between first and last visit (logMAR)	0.0 (−0.3, 0.2)	−0.65 (−1.5, −0.2)	**0.001**

*Mann–Whitney U test (significance, *p* < 0.05).

^b^BCVA within 6 months of onset.

BCVA: best-corrected visual acuity; IQR, interquartile range.

The associations between gender, mutation type, age, and BCVA values at the first and last visits were determined (Table 4). We found that the G11778A mutation, male gender, and an older age were related to a worsened BCVA (*P* values < .001, .001, and .004, respectively). However, none of those three factors were associated with the BCVA at the first visit, based on the patients in this review.

**Table 4.** Generalised estimating equation (GEE) analysis of correlation between final BCVA and other variables.

Variables	Co-efficiency	Standard error	95% CI	*P* value
Male gender	1.255	0.333	0.602 to 1.908	**<0.001**
G11778A mutation	1.016	0.297	0.434 to 1.598	**0.001**
Age	0.033	0.011	0.010 to 0.055	**0.004**
BCVA (logMAR) at first visit	0.014	0.135	−0.250 to 0.278	0.918

BCVA: best-corrected visual acuity.

## Discussion

This retrospective chart review demonstrated the epidemiology of a population of Thai patients had been diagnosed with LHON between 1997 and 2016. The study enrolled 229 patients diagnosed by clinical and genetic testing of three common mutations. The G11778A mutation represented the majority (91%), while T14484C accounted for the remainder. Our review did not detect any cases of the G3460A mutation. We compared the group characteristics of the patients with the two types of mutation. We found that the median age at G11778A onset (21.9 years) was approximately 10 years less than that for T14484C. Almost all patients were males. At baseline, there was no significant difference in the visual losses of the patients with the G11778A and T14484C mutations. However, there was a distinct gap in the degree of visual losses by the final follow-up visit. On average, the patients with the T14484C mutation demonstrated considerable recovery of vision, whereas those with the G11778A mutation did not deviate from their baseline BCVA. An analysis of the clinical, demographic, and genetic-testing data revealed that the G11778A mutation, male gender, and an older age at onset were associated with a poor prognosis.

Overall, our results correspond with those of several studies in Asia [13,14]. They reported that G11778A is the most frequently found LHON mtDNA mutation whereas—unlike the situation for people of European and Western Siberia descent—G3460A is rarely found [1,4,15,16]. The prevalence of G3460A is much higher in Western Siberia than Europe, whereas the prevalence of G11778A in Western Siberia is lower than in both Europe and Asia [16]. This variation among ethnicities raises the question of what the underlying mechanism is. One possible answer relates to the genetic variation among lineages. Many studies have revealed how specific and common mitochondrial haplogroup J is in G11778A and T14484C LHON patients [17,18]. Further studies identified haplogroup’s association with toxic environment susceptibility and its high penetrance characteristic [19,20]. Contrary to expectations, haplogroup J was not obvious in the Asian population. Instead, in studies on LHON patients in Thailand [11] and in China, [21] mitochondrial haplogroups M, B*, and B demonstrated high frequencies, whereas mitochondrial haplogroup F showed a low frequency. In addition, a study by Sudoyo and colleagues [10] found many novel single-nucleotide polymorphisms were linked to mitochondrial haplogroup M in Southeast Asian LHON patients.

This study showed the higher male to female ratio and lower prevalence of blood heteroplasmy compared to the previous study [22]. However, a number and variety of patients included in the current study were more than the previous one as we recruited all LHON patients across the country, covering both the G11778A and T14484C mutation. A lack of awareness of LHON in females may have contributed to the surprisingly low number of diagnoses for women in this study. One example recently found at our clinic was a female who had previously been diagnosed with neuromyelitis optica spectrum disorder, based on a visual loss and limb weakness. A bilateral cecocentral scotoma defect detected in a visual field review raised suspicion of LHON, and subsequent genetic testing revealed a G11778A mutation. A large international study of LHON demographics showed a higher prevalence of female than earlier studies and assumed that there might be an ascertainment bias in favour of the disease affecting young men [23].

The median age at onset in our study did not deviate from existing data, which indicates that the age at presentation is predominantly in the 15- to 35-year-old age range, particularly with males. Nevertheless, it is not surprising that the oldest case in our study presented at an age over 60 years: such an age is still consistent with the findings of several earlier studies [14,23].

From our experience, LHON patients with visual-acuity improvement tend to become lost to follow-up or decide to attend clinics that are closer to their residences. It was therefore not surprising that the follow-up period of the patients with the T14485C mutation was not as lengthy as that of the patients with the G11778A mutation. Furthermore, a few studies [24,25] demonstrated that the X-linked modifier loci of LHON is a gender factor that may be related to the earlier and more severe visual loss that is found in males compared with females. Finally, the chances of restoring vision are much higher for childhood-onset LHON, [26,27] regardless of the mutation type. Thus, it raises the possibility that a younger age is a positive factor for LHON.

The international consensus statement on the clinical and therapeutic management of LHON published in 2017, [28] recommended to treat with idebenone 900 mg/day for at least a year to assess the therapeutic response. Unfortunately, this study included patients prior to 2017. Therefore, curcumin, coenzyme Q10, idebenone, vitamin B, multivitamins, and steroids were used to treat our patients. The dosage and duration of treatment were both variable. As a result, it was unable to exactly compare and analyse the treatment outcomes. Moreover, idebenone is not available in Thailand and most of the patients cannot afford it.”

## Limitations

Due to the study design (retrospective chart review), measurements other than BCVA were inconsistent and hard to collect for analysis. Moreover, financial constraints historically prevented mutation testing being conducted for all cases of suspected LHON, and asymptomatic family members were generally not investigated for the same reason. All of these factors meant that the present study could not capture complete genealogical data and pedigrees, and it proved difficult to determine the true incidence and prevalence of LHON in Thailand. Moreover, due to the very low number of female LHON patients, we were unable to clarify the clinical features that may be related to gender, for instance, age distribution.

## Conclusions

This is the first epidemiological study of LHON patients in Thailand, and it provides data relating to Southeast Asia for the first time. The G11778A missense is the leading mutation in Thai LHON patients, followed by the T14484C mutation. Our review did not find any of the G3460A mutations in the study population. The vast majority of the Thai LHON patients were male and presented as young adults. There were only a few patients who had facioscapulohumeral muscular dystrophy with G11778A mutation. The G11778A mutation, an older age, and the male gender were related to poor vision recovery.

## Data Availability

The data that support the findings of this study are available from the corresponding author, NC, upon reasonable request.
